# Checklist of acanthocephalan parasites of South Africa

**DOI:** 10.3897/zookeys.789.27710

**Published:** 2018-10-10

**Authors:** Ali Halajian, Lesley R. Smales, Sareh Tavakol, Nico J. Smit, Wilmien J. Luus-Powell

**Affiliations:** 1 DST-NRF SARChI Research Chair (Ecosystem Health), Department of Biodiversity, University of Limpopo, Sovenga, 0727, South Africa University of Limpopo Sovenga South Africa; 2 Parasitology Section, South Australia Museum, North Terrace, Adelaide 5000 South Australia, Australia South Australia Museum Adelaid Australia; 3 Water Research Group, Unit for Environmental Sciences and Management, North-West University, Potchefstroom, South Africa North-West University Potchefstroom South Africa

**Keywords:** Acanthocephala, helminths, diversity, wildlife, Africa

## Abstract

Twenty-one species of acanthocephalans, representative of thirteen genera from ten families of seven orders and three classes, are included in this updated checklist of acanthocephalans in South Africa.

Although South Africa appears to have a less diverse acanthocephalan fauna compared to some other countries such as Iran in Asia, or Brazil in South America, this is probably an artefact of fewer parasitological surveys.

## Introduction

South Africa’s landscape is the third most biologically diverse in the world with 6% of the world’s mammal species, 8% of bird species and 5% of reptile species of which many are endemic (da Silva and Willows-Munro 2016). In regard to invertebrates, only 36,803 species are listed for Africa, and 12,098 for South Africa (Hamer 2010). It is said that in South African habitats, there are many undiscovered and undescribed animal species, especially invertebrates. It is estimated that as many as 80,000 South African animal species remain to be discovered or described, and most of these animals are invertebrates (Hamer 2013).

The most comprehensive checklist of helminth parasites of Africa was compiled for freshwater fishes by Khalil (1971) and updated by Khalil and Polling (1997). It included very few records of acanthocephalans considering the total number of freshwater fish species present in the continent. The updated list (Khalil and Polling 1997) comprised 568 adult helminth parasites of which only 21 species are acanthocephalans. These records were compiled from 359 species of African freshwater fishes (assigned to 89 genera belonging to 32 families) of an estimated 3000 existing inland fish species (Khalil and Polling 1997). These examples illustrate the lack of knowledge of the helminth fauna of the wildlife of the African continent in general and in South Africa in particular.

This is the first checklist of acanthocephalans of South Africa and the aim is to provide a comprehensive record of all the previously reported species of Acanthocephala occurring in South African hosts as well as new records from our on-going research on parasites of wildlife, while simultaneously demonstrating the need for more extensive parasitological surveys.

## Materials and methods

Data were obtained from two sources, published records and our own ongoing studies on the Acanthocephala of South African wildlife. These data are presented in two parts. In the first part parasites are listed systematically, with families, genera, and species in alphabetical order. The scientific name, including any synonyms, followed by the scientific and common name of the host, the locality in which the parasite was reported and museum (location) of type specimens where known. In the second part, the hosts are listed systematically by their scientific names and parasite records from each host are given together with locality and reference. The records without references are those of our ongoing study that are being reported here for the first time.

Classification of the Acanthocephala follows Amin (2013). For the hosts, fish taxonomy is based on Skelton (2001, 2016) and Fishbase (Froese and Pauly 2016), bird taxonomy is based on Clements et al. (2016) and mammal taxonomy on Wilson and Reader (2005) and Apps (2012).

Abbreviations for museums are:

**BMNH**Natural History Museum London, London, UK;

**GNM**Gothenburg Natural History Museum, Gothenburg, Sweden;

**SAMCTA**South African Museum at Cape Town, South Africa;

**SAM**South Australian Museum, Adelaide, Australia;

**USNM Helm. Coll.** United States National Museum Helminthological Collection

**USNPC**United States National Parasite Collection now held in the Invertebrate Zoology collection of the Smithsonian Museum, Washington, USA.

Acanthocephalan specimens from our ongoing wildlife parasitology projects were mostly collected from roadkill animals, museum collections, hunting/culling surveys and other research permits received for a limited number of specimens through the Limpopo Department of Economic Development, Environment and Tourism (LEDET) (permit number CPM004961 and ZA/LP/HO/3370 for freshwater fish research, 001-CPM403-00012 and ZA/LP/HO/3448 for frogs, ZA/LP/HO/3432 for rodents and ZA/LP/87586 for roadkills).

Acanthocephalans from freshly dead animals were placed in tap water and refrigerated for a few hours to one day until the proboscis was everted and then fixed and stored in 70% ethanol until studied. Acanthocephalans from frozen hosts were fixed and stored in 70% ethanol. Some specimens were prepared for examination by staining in Mayer’s acid carmine, destained in HCl in 70% ethanol, dehydrated through increasing concentrations of ethanol, cleared in xylene, and mounted as whole worms in Canada balsam. Other worms were examined as temporary mounts following clearing in lactophenol or beechwood creosote.

A total of 102 species of birds (151 individuals), 72 of mammals (420 individuals), 9 of reptiles (18 individuals) and 42 (1050 individuals) of fishes were examined for this study (details in Table 1).

**Table 1.** Total number of host taxa examined and those infected with acanthocephalans (i.e. number of taxa that harboured acanthocephalans, in parenthesis) during our ongoing study on wildlife parasites in South Africa.

Taxon Group	Order	Family	Genus	Species
**Amphibians**	1 (0)	8 (0)	13 (0)	19 (0)
**Birds**	21 (5)	50 (5)	87 (5)	102 (5)
**Fishes** (freshwater)	8 (1)	13 (1)	24 (1)	42 (1)
**Mammals**	10 (4)	26 (4)	59 (6)	72 (6)
**Reptiles**	3 (1)	6 (1)	7 (1)	9 (1)
**Totals**	43 (11)	103 (11)	190 (13)	244 (13)

## Parasite-Host List

### Acanthocephala

#### Class: Archiacanthocephala Meyer, 1931

##### Order: Gigantorhynchida Southwell & Macfie, 1925

###### Family: Gigantorhynchidae Hamann, 1892

####### Genus: *Mediorhynchus* Van Cleave, 1916

######## Mediorhynchus africanus

Taxon classificationAnimaliaGigantorhynchidaGigantorhynchidae

Amin, Evans, Heckmann & El-Naggar, 2013

Empodius segmentatus (de Marval, 1902) Southwell & Mac-Fie, 1925
Mediorhynchus selengensis Harris, 1973
M. gallinarum (Bhalerao, 1937) Van Cleave, 1947 *sensu* Junker & Boomker, 2006

######### Notes.

*M.gallinarum* is found only in Asia and the species in Africa is actually *M.africanus* (Amin et al. 2013; Amin 2013) and not *M.gallinarum* previously reported in South Africa (Junker and Boomker 2006).

######### Host.

*Numidameleagris* (L. 1758) (Helmeted Guineafowl) (Numididae) (type host).

######### Localities.

Kruger National Park, Mpumalanga Province, South Africa (type locality) (Junker and Boomker 2006); Vicinity of Petrus Steyn, Free State Province, South Africa (Davies et al. 2008); Musina, Limpopo Province, South Africa (Junker and Boomker 2007; Junker et al. 2008).

######## Mediorhynchus mokgalongi

Taxon classificationAnimaliaGigantorhynchidaGigantorhynchidae

Smales & Halajian, 2018

######### Host.

*Turdussmithi* Bonaparte, 1850 (Karoo Thrush) (Turdidae) (type host).

######### Locality.

Polokwane, Limpopo Province, South Africa (type locality) (Smales et al. 2018).

######### Type specimens.

Holotype male SAM AHC 48068, allotype female SAM AHC 48069, paratype SAM AHC 48070.

######## Mediorhynchus numidae

Taxon classificationAnimaliaGigantorhynchidaGigantorhynchidae

(Baer, 1925) Meyer, 1932

Heteroplus numidae Baer, 1925; Empodismanumidae (Baer, 1925) Yamaguti, 1963

######### Host.

*Numidameleagris* (L. 1758) (Helmeted Guineafowl) (Numididae)

######### Locality.

Pretoria, Gauteng Province, South Africa (Oosthuizen and Markus 1967).

######## Mediorhynchus taeniatus

Taxon classificationAnimaliaGigantorhynchidaGigantorhynchidae

(von Linstow, 1901) Dollfus, 1936

Echinorhynchus taeniatus von Linstow, 1901; E.segmentatus de Marval, 1902

######### Host.

*Numidameleagris* (L. 1758) (Helmeted Guineafowl) (Numididae).

######### Locality.

Rooipoort farm, Kimberley, Northern Cape Province, South Africa (Crowe 1977).

######### Host.

*Tockusleucomelas* (Lichtenstein, 1842) (Southern Yellow-billed Hornbill) (Bucerotidae)

######### Locality.

Limpopo Province, South Africa.

##### Order: Moniliformida Schmidt, 1972

###### Family: Moniliformidae Van Cleave, 1924

####### Genus: *Moniliformis* Travassos, 1915

######## Moniliformis kalahariensis

Taxon classificationAnimaliaMoniliformidaMoniliformidae

Meyer, 1931

######### Host.

*Atelerixfrontalis* (Smith, 1831) (Southern African Hedgehog) (Erinaceidae)

######### Locality.

Mohlonong village and University of Limpopo, Limpopo Province, South Africa (Amin et al. 2014).

######## Moniliformis moniliformis

Taxon classificationAnimaliaMoniliformidaMoniliformidae

(Bremser, 1811) Travassos, 1915 (type species)

Echinorhynchus moniliformis Bremser, 1811
E. grassi Railliet, 1893
E. canis Porter, 1914
E. belgicus Railliet, 1919
Moniliformis moniliformis aegypticus Meyer in Petrochenko, 1958
M. dubius Meyer, 1932
M. travassosi Meyer, 1932 (*fide* Machado Filho 1946, Van Cleave 1952)

######### Host.

*Atelerixfrontalis* (Smith, 1831) (Southern African Hedgehog) (Erinaceidae).

######### Locality.

Hammanskraal, Gauteng Province, South Africa (Le Roux 1930).

######### Notes.

Host recorded as *Aethechinusfrontalis* in Le Roux (1930).

######## Moniliformis acomysi

Taxon classificationAnimaliaMoniliformidaMoniliformidae

Ward & Nelson, 1967

######### Host.

*Gerbilliscusleucogaster* (Peters, 1852) (Bushveld Gerbil), *Mastomysnatalensis* (Smith, 1834) (Natal Mastomys), *Musminutoides* (Pygmy mouse) (Muridae).

######### Localities.

Bloemhof, Free State Province; Vyeboom village, Limpopo Province; Hoopstad, Free State Province; South Africa.

######## Moniliformis

Taxon classificationAnimaliaMoniliformidaMoniliformidae

sp.

######### Host.

*Otolemurcrassicaudatus* (É. Geoffroy Saint-Hilaire, 1812) (Thick-tailed Bushbaby) (Galagidae).

######### Locality.

Venda, Limpopo Province, South Africa.

######### Remarks.

One male and one female worm were found in the small intestine of an adult bushbaby.

##### Order: Oligacanthorhynchida Petrochenko, 1956

###### Family: Oligacanthorhynchidae Southwell & Macfie, 1925

####### Genus: *Heptamegacanthus* Spencer Jones, 1990

######## Heptamegacanthus niekerki

Taxon classificationAnimaliaOligacanthorhynchidaOligacanthorhynchidae

Spencer Jones, 1990 (type species)

######### Host.

*Chrysospalaxtrevelyani* (Günther, 1875) (Giant Golden Mole) (Chrysochloridae) (type host).

######### Locality.

Nqadu Forest, Transkei, Eastern Cape Province, South Africa (type locality) (Spencer Jones 1990).

######### Type specimens.

Holotype male BMNH 1988.2480; allotype female BMNH 1988.2481; paratypes BMNH 1988.2482-2491.

######## Oligacanthorhynchidae

Taxon classificationAnimaliaOligacanthorhynchidaOligacanthorhynchidae

sp.

######### Host.

*Varanusalbigularis* Daudin, 1802 (Rock Monitor) (Varanidae).

######### Localities.

Tzaneen; Tolwe, Limpopo Province, South Africa.

#### Class: Eoacanthocephala Van Cleave, 1936

##### Order: Gyracanthocephala Van Cleave, 1936

###### Family: Quadrigyridae Van Cleave, 1920

####### Genus: *Acanthogyrus* Thapar, 1927

######## Subgenus: *Acanthosentis* Verma & Datta, 1929

######### Acanthogyrus (Acanthosentis) phillipi

Taxon classificationAnimaliaGyracanthocephalaQuadrigyridae

(Mashego, 1988) Amin, 2005

Acanthosentisphillipi Mashego, 1988

########## Host.

*Enteromiusneefi* (Greenwood, 1962) (syn. *Barbusneefi*) (Sidespot Barb) (Cyprinidae) (type host).

########## Locality.

Lingwe River, Venda, Limpopo Province, South Africa (type locality) (Mashego 1988).

########## Type specimens.

Holotype in Transvaal Museum no. TM14659; Paratypes TM5 at University of Limpopo, Zoology, 5.

######### Acanthogyrus

Taxon classificationAnimaliaGyracanthocephalaQuadrigyridae

sp.

########## Host.

*Oreochromismossambicus* (Peters, 1852) (Mozambique Tilapia) (Cichlidae).

########## Locality.

Molepo Dam, Limpopo Province, South Africa (Kunutu et al. 2013).

##### Order: Neoechinorhynchida Southwell & Macfie, 1925

###### Family: Neoechinorhynchidae (Ward, 1917) Van Cleave, 1928

####### Genus: *Neoechinorhynchus* Stiles & Hassall, 1905

######## Subgenus: *Neoechinorhynchus* Hamann, 1892

######### Neoechinorhynchus (Neoechinorhynchus) dorsovaginatus

Taxon classificationAnimaliaNeoechinorhynchidaNeoechinorhynchidae

Amin & Christison, 2005

########## Host.

*Argyrosomusjaponicus* (Temminck & Schlegel, 1843) (Japanese Meagre, Dusky Kob) Sciaenidae) (type host).

########## Locality.

Breede River Estuary, Western Cape Province, South Africa (type locality) (Amin and Christison 2005).

########## Type specimens.

No. SAMCTA29536 (holotype male and allotype female; same slide), nos SAMCTA29537-29545 (paratypes), USNPC no. 94918 (paratypes).

#### Class: Palaeacanthocephala Meyer, 1931

##### Order: Echinorhynchida Southwell & Macfie, 1925

###### Family: Pomphorhynchidae Yamaguti, 1939

####### Genus: *Longicollum* Yamaguti, 1935

######## Longicollum chabanaudi

Taxon classificationAnimaliaEchinorhynchidaPomphorhynchidae

Dollfus & Golvan, 1963

######### Host.

*Barnardichthysfulvomarginata* (Gilchrist, 1904) (syn. *B.fulvomarginatus*) (Soleidae) (Sole).

######### Locality.

False Bay, Western Cape Province, South Africa (Dollfus and Golvan 1963).

######## Longicollum

Taxon classificationAnimaliaEchinorhynchidaPomphorhynchidae

sp. innom.

######### Host.

*Pegusanasuta* (Pallas, 1814) (syn. *Soleableekeri*) (Blackhand Sole) (Soleidae).

######### Locality.

Klein River estuary, Hermanus, Western Cape Province, South Africa (Bray 1974).

######### Remarks.

Three contracted and immature specimens were found in one fish and one male in another fish. Thus it could not be identified to species level (Bray 1974).

###### Family: Rhadinorhynchidae Lühe, 1912

####### Genus: *Rhadinorhynchus* Lühe, 1911

######## Rhadinorhynchus cadenati

Taxon classificationAnimaliaEchinorhynchidaRhadinorhynchidae

(Golvan & Houin, 1964) Golvan, 1969

Nipporhynchus cadenati Golvan & Houin, 1964

######### Host.

*Thyrsitesatun* (Euphrasen, 1791) (Snoek) (Gempylidae).

######### Locality.

South Africa’s West and South coasts (Nunkoo et al. 2016).

######## Rhadinorhynchus capensis

Taxon classificationAnimaliaEchinorhynchidaRhadinorhynchidae

Bray, 1974

######### Host.

*Pegusanasuta* (Pallas, 1814) (syn. *Soleableekeri*) (Blackhand Sole) (Soleidae) (type host)

######### Locality.

Heuninges River estuary, near Cape Agulhas, Western Cape Province, South Africa (type locality) (Bray 1974).

######### Type specimens.

British Museum, Registration number 1974.521-550.

######## Rhadinorhynchus

Taxon classificationAnimaliaEchinorhynchidaRhadinorhynchidae

sp.

######### Host.

*Ruvettuspretiosus* Cocco, 1833 (Oilfish) (Gempylidae).

######### Locality.

South Africa’s West coast, Atlantic Ocean (Nunkoo et al. 2017).

##### Order: Polymorphida Petrochenko, 1956

###### Family: Centrorhynchidae Van Cleave, 1916 (Golvan, 1960)

####### Genus: *Centrorhynchus* Lühe, 1911

######## Centrorhynchus sarehae

Taxon classificationAnimaliaPolymorphidaCentrorhynchidae

Smales & Halajian, 2017

######### Host.

*Kaupifalcomonogrammicus* (Temminck, 1824) (Lizard Buzzard) (Accipitridae) (type host).

######### Locality.

Makhado (Louis Trichardt), Limpopo Province, South Africa (type locality) (Smales et al. 2017).

######### Type Specimens.

Holotype male SAM AHC 47858; allotype female SAM AHC 47859; paratypes SAM AHC 47860.

######## Centrorhynchus clitorideus

Taxon classificationAnimaliaPolymorphidaCentrorhynchidae

(Meyer, 1931) Golvan, 1958

Gordiorhynchus clitorideus Meyer, 1931 (*nec clitorideum*)

######### Host.

*Buboafricanus* (Temminck, 1821) (Spotted Eagle Owl) (Strigidae).

######### Locality.

Zandrivierspoort Farm, Polokwane, Limpopo Province, South Africa.

######## Centrorhynchus

Taxon classificationAnimaliaPolymorphidaCentrorhynchidae

sp.

######### Host.

*Feliscatus* L., 1758 (Domestic Cat) (Felidae).

######### Locality.

Pretoria, Gauteng Province, South Africa (Baker et al. 1989).

######## Centrorhynchus

Taxon classificationAnimaliaPolymorphidaCentrorhynchidae

sp.

######### Host.

*Mungosmungo* (Gmelin, 1788) (Banded Mongoose) (Herpestidae).

######### Locality.

Polokwane, Limpopo Province, South Africa.

###### Family: Plagiorhynchidae Golvan, 1960

####### Genus: *Plagiorhynchus* Lühe, 1911

######## Subgenus: *Prosthorhynchus* Kostylew, 1915

######### Plagiorhynchus (Prosthorhynchus) cylindraceus

Taxon classificationAnimaliaPolymorphidaPlagiorhynchidae

(Goeze, 1782) Schmidt & Kuntz, 1966

Echinorhynchus cylindraceus Goeze, 1782
E. pici Gmelin, 1791 *fide* Florescu and Ienistea 1984
E. merulae Gmelin, 1791 *fide* Florescu and Ienistea 1984
E. transversus (Rudolphi, 1819) Travassos 1926
E. obliquus Dujardin, 1845 *fide* Florescu and Ienistea 1984
Centrorhynchus cylindraceus (Goeze 1782) Kostylew, 1914
C. fasciatus (Westrumb, 1821) Travassos, 1926 *fide* de Marval 1905
C. rostratus de Marval, 1902 *fide* Florescu and Ienistea 1984
Prosthorhynchus rosai (Porta, 1910) Meyer, 1932
Prosthorhynchus rostratus (de Marval, 1902) Meyer, 1932
Plagiorhynchus formosus Van Cleave, 1918 *fide*Amin et al. 1999
Plagiorhynchus taiwanensis Schmidt et Kuntz, 1966 *fide*Amin et al. 1999.

########## Host.

*Calidrisferruginea* (Pontoppidan, 1763) (Curlew Sandpiper); *Charadriuspecuarius* Temminck, 1823 (Kittlitz’s Plover); *Charadriustricollaris* Vieillot, 1818 (Three-banded Plover) (Charadriidae).

########## Locality.

Berg River, Western Cape Province, South Africa (Amin et al. 1999).

########## Host.

*Crecopsisegregia* (Peters, 1854) (syn. *Crexegregia*) (African Crake) (Rallidae).

########## Locality.

Blouberg, Limpopo Province, South Africa.

########## Host.

*Vanellusarmatus* (Burchell, 1822) (Blacksmith Lapwing) (Charadriidae).

########## Locality.

Berg River, Western Cape Province, South Africa (Amin et al. 1999).

######### Unidentified plagiorhynchid

**Host.***Charadriusmarginatus* Vieillot, 1818 (White-fronted Plover), *Charadriuspallidus* Strickland, 1852 (Chestnut-banded Plover); *Charadriuspecuarius* Temminck, 1823 (Kittlitz’s Plover); *Himantopushimantopus* (L., 1758) (Black-winged Stilt); *Vanellusarmatus* (Burchell, 1822) (Blacksmith Lapwing)

**Locality.** Berg River, Western Cape Province, South Africa (Amin et al. 1999).

###### Family: Polymorphidae Meyer, 1931

####### Genus: *Andracantha* Schmidt, 1975

######## Andracantha tunitae

Taxon classificationAnimaliaPolymorphidaPolymorphidae

(Weiss, 1914) Zdzitowiecki, 1989

Corynosoma tunitae Weiss, 1914

######### Host.

*Microcarboafricanus* (Gmelin, 1789) (Long-tailed Cormorant) (Phalacrocoracidae).

######### Locality.

Dyer Island, South Africa (Van Cleave 1937).

######### Notes.

Van Cleave (1937) is mentioning that he looked at a large number of immature worms and he tentatively assigning them to *C.tunitae*.

####### Genus: *Arhythmorhynchus* Lühe, 1911

*Skrjabinorhynchus* Petrochenko, 1956

######## Arhythmorhynchus turbidus

Taxon classificationAnimaliaPolymorphidaPolymorphidae

(Van Cleave, 1937) Golvan, 1994

Corynosoma turbidum Van Cleave, 1937

######### Host.

*Phalacrocoraxneglectus* (Wahlberg, 1855) (Bank Cormorant) (Phalacrocoracidae) (type host).

######### Locality.

Dyer Island, South Africa (type locality) (Van Cleave 1937).

######### Type specimens.

Holotype female (1737, 3) and one paratype female (1737, 1) in GNM. One paratype female (1737, 2) in the collection of H.J. Van Cleave, Urbana, Illinois, U.S.A.

####### Genus: *Bolbosoma* Porta, 1908

######## Bolbosoma capitatum

Taxon classificationAnimaliaPolymorphidaPolymorphidae

(von Linstow, 1880) Porta, 1908

Echinorhynchus capitatum von Linstow, 1880
Bolbosoma physeteris Gubanov, 1952 (*fide* Amin & Margolis, 1998)

######### Host.

*Ruvettuspretiosus* Cocco, 1833 (Oilfish) (Gempylidae).

######### Locality.

South Africa’s West coast, Atlantic Ocean (Nunkoo et al. 2017).

######## Bolbosoma vasculosum

Taxon classificationAnimaliaPolymorphidaPolymorphidae

(Rudolphi, 1819) Porta, 1908

Echinorhynchus vasculosum Rudolphi, 1819
Bolbosoma annulatus Molin, 1858
B. aurantiacus Risso, 1826
B. pellucidus Leukart, 1828
B. serrani Linton, 1888
B. thunni Harada, 1935 (*fide* Petrochenko 1958)

######### Host.

*Thyrsitesatun* (Euphrasen, 1791) (Snoek) (Gempylidae).

######### Locality.

South Africa’s West and South coasts (Nunkoo et al. 2016).

####### Genus: *Corynosoma* Lühe, 1904 (*fide* Van Cleave, 1945)

*Chentrosoma* Monticelli, 1905

*Centrosoma* Lühe, 1912

*Coryusoma* Railliet & Henry, 1907 (misprint)

*Echinosoma* Porta, 1907

######## Corynosoma australe

Taxon classificationAnimaliaPolymorphidaPolymorphidae

Johnston, 1937

Corynosoma otariae Morini & Boero, 1961

######### Host.

*Thyrsitesatun* (Euphrasen, 1791) (Snoek) (Gempylidae).

######### Locality.

South Africa’s West and South coasts (Nunkoo et al. 2016).

## Host-Parasite List

**Table:** 

Host	Parasite	Locality Country/ Reference	
**Class Aves**			
**Order Accipitriformes**			
**Family Accipitridae**			
*Kaupifalcomonogrammicus* (type host)	*Centrorhynchussarehae* (Centrorhynchidae)	Makhado (Louis Trichardt), Limpopo Province, South Africa	
**Order Bucerotiformes**			
**Family Bucerotidae**			
* Tockus leucomelas *	*Mediorhynchustaeniatus* (Gigantorhynchidae)	Limpopo Province, South Africa	
**Order Charadriiformes**			
**Family Charadriidae**			
* Calidris ferruginea *	Plagiorhynchus (Prosthorhynchus) cylindraceus (Plagiorhynchidae)	Berg River, Western Cape Province, South Africa (Amin et al. 1999)	
* Charadrius marginatus *	Unidentified plagiorhynchid acanthocephalan (Plagiorhynchidae)	Berg River, Western Cape Province, South Africa (Amin et al. 1999)	
* Charadrius pallidus *	Unidentified plagiorhynchid acanthocephalan (Plagiorhynchidae)	Berg River, Western Cape Province, South Africa (Amin et al. 1999)	
* Charadrius pecuarius *	Plagiorhynchus (Prosthorhynchus) cylindraceus (Plagiorhynchidae)	Berg River, Western Cape Province, South Africa (Amin et al. 1999)	
	Unidentified plagiorhynchid acanthocephalan (Plagiorhynchidae)	Berg River, Western Cape Province, South Africa (Amin et al. 1999)	
* Charadrius tricollaris *	Plagiorhynchus (Prosthorhynchus) cylindraceus (Plagiorhynchidae)	Berg River, Western Cape Province, South Africa (Amin et al. 1999)	
* Vanellus armatus *	Plagiorhynchus (Prosthorhynchus) cylindraceus (Plagiorhynchidae)	Berg River, Western Cape Province, South Africa (Amin et al. 1999)	
	Unidentified plagiorhynchid acanthocephalan (Plagiorhynchidae)	Berg River, Western Cape Province, South Africa (Amin et al. 1999)	
**Family Recurvirostridae**			
* Himantopus himantopus *	Unidentified plagiorhynchid Acanthocephalan (Plagiorhynchidae)	Berg River, Western Cape Province, South Africa (Amin et al. 1999)	
**Order Galliformes**			
**Family Numididae**			
*Numidameleagris* (type host)	*Mediorhynchusafricanus* (previously identified as *Mediorhynchusgallinarum*) (Gigantorhynchidae)	Kruger National Park, Mpumalanga Province, South Africa (Junker and Boomker 2006); Vicinity of Petrus Steyn, Free State Province, South Africa (Davies et al. 2008); Musina, Limpopo Province, South Africa (Junker and Boomker 2007; Junker et al. 2008)	
	*Mediorhynchusnumidae* (Gigantorhynchidae)	Pretoria, Gauteng Province, South Africa (Oosthuizen and Markus 1967)	
	*Mediorhynchustaeniatus* (Gigantorhynchidae)	Rooipoort farm, Kimberley, Northern Cape Province, South Africa (Crowe 1977)	
**Order Gruiformes**			
**Family Rallidae**			
* Crecopsis egregia *	Plagiorhynchus (Prosthorhynchus) cylindraceus (Plagiorhynchidae)	Blouberg, Limpopo Province, South Africa	
**Order Passeriformes**			
**Family Turdidae**			
*Turdussmithi* (type host)	*Mediorhynchusmokgalongi* (Gigantorhynchidae)	Polokwane, Limpopo Province, South Africa (type locality) (Smales et al. 2018)	
**Order Strigiformes**			
**Family Strigidae**			
* Bubo africanus *	*Centrorhynchusclitorideus* (Centrorhynchidae)	Zandrivierspoort Farm, Polokwane, Limpopo Province, South Africa	
**Order Suliformes**			
**Family Phalacrocoracidae**			
* Microcarbo africanus *	*Andracanthatunitae* (Polymorphidae)	Dyer Island, South Africa (Van Cleave 1937)	
*Phalacrocoraxneglectus* (type host)	*Arhythmorhynchusturbidus* (Polymorphidae)	Dyer Island, South Africa (type locality) (Van Cleave 1937)	
**Class Actinopterygii**			
**Order Cypriniformes**			
**Family Cyprinidae**			
* Enteromius neefi *	Acanthogyrus (Acanthosentis) phillipi (Quadrigyridae)	Lingwe River, Venda, Limpopo Province, South Africa (Mashego 1988)	
**Order Perciformes**			
**Family Cichlidae**			
* Oreochromis mossambicus *	*Acanthogyrus* sp. (Quadrigyridae)	Molepo dam, Limpopo Province, South Africa (Kunutu et al. 2013)	
**Family Gempylidae**			
* Ruvettus pretiosus *	*Bolbosomacapitatum* (Polymorphidae)	South Africa’s West coast, Atlantic Ocean (Nunkoo et al. 2017)	
	*Rhadinorhynchus* sp. (Rhadinorhynchidae)	South Africa’s West coast, Atlantic Ocean (Nunkoo et al. 2017)	
* Thyrsites atun *	*Bolbosomavasculosum* (Polymorphidae)	South Africa’s West and South coasts (Nunkoo et al. 2016)	
	*Corynosomaaustrale* (Polymorphidae)	South Africa’s West and South coasts (Nunkoo et al. 2016)	
	*Rhadinorhynchuscadenati* (Rhadinorhynchidae)	South Africa’s West and South coasts (Nunkoo et al. 2016)	
**Family Sciaenidae**			
*Argyrosomusjaponicus* (type host)	Neoechinorhynchus (Neoechinorhynchus) dorsovaginatus (Neoechinorhynchidae)	Breede River Estuary, Western Cape Province, South Africa (type locality) (Amin and Christison 2005)	
**Order Pleuronectiformes**			
**Family Soleidae**			
*Pegusanasuta* (*Soleableekeri*) (type host)	*Rhadinorhynchuscapensis* (Rhadinorhynchidae)	Heuninges River estuary (Bray 1974)	
	*Longicollum* sp. innom. (Pomphorhynchidae)	Klein River estuary (Bray 1974)	
*Barnardichthysfulvomarginata* (type host)	*Longicollumchabanaudi* (Pomphorhynchidae)	False Bay, Western Cape Province (Dollfus and Golvan 1963)	
**Class Mammalia**			
**Order Afrosoricida**			
**Family Chrysochloridae**			
*Chrysospalaxtrevelyani* (type host)	*Heptamegacanthusniekerki* (Oligacanthorhynchidae)		Nqadu Forest, Transkai, Eastern Cape Province, South Africa (Spencer Jones 1990)
**Order Carnivora**			
**Family Felidae**			
* Felis catus *	*Centrorhynchus* sp. (Centrorhynchidae)		Pretoria, Gauteng Province, South Africa (Baker et al. 1989)
**Family Herpestidae**			
* Mungos mungo *	*Centrorhynchus* sp. (Centrorhynchidae)		Polokwane, Limpopo Province, South Africa.
**Order Eulipotyphla**			
**Family Erinaceidae**			
*Atelerixfrontalis* (*Aethechinusfrontalis*)	*Moniliformiskalahariensis* (Moniliformidae)		Mohlonong village and University of Limpopo, Limpopo Province, South Africa (Amin et al. 2014)
	*Moniliformismoniliformis* (Moniliformidae)		Hammanskraal, Gauteng Province, South Africa (Le Roux 1930)
**Order Primates**			
**Family Galagidae**			
* Otolemur crassicaudatus *	*Moniliformis* sp. (Moniliformidae)		Venda, Limpopo Province, South Africa
**Order Rodentia**			
**Family Muridae**			
* Gerbilliscus leucogaster *	*Moniliformisacomysi* (Moniliformidae)		Vyeboom village, Limpopo Province, South Africa
* Mastomys natalensis *	*Moniliformisacomysi* (Moniliformidae)		Bloemhof, Free State Province
* Mus minutoides *	*Moniliformisacomysi* (Moniliformidae)		Hoopstad, Free State Province, South Africa
**Class Reptilia**			
**Order Squamata**			
**Family Varanidae**			
* Varanus albigularis *	Oligacanthorhynchidae sp. (Oligacanthorhynchidae)		Tzaneen; Tolwe, Limpopo Province, South Africa

## Results and discussion

A total of twenty-one species of acanthocephalans, from thirteen genera from ten families of seven orders, comprise this updated checklist of acanthocephalans in South Africa. Representatives of three of the four classes of acanthocephalans (Amin 2013) have been reported in South Africa, with only the Polyacanthocephala Amin, 1987 not having been recorded yet. The composition of reported acanthocephalan fauna shows that the Polymorphidae is the most represented family with five named species parasitic in marine fish and wild birds.

In South Africa, birds have the highest species richness of acanthocephalans to this date with nine named species (from five genera) and five records only identified to group level, followed by fish with eight named species (from six genera) and two species only identified to genus level, mammals with four named species (from two genera) and three species only identified to genus level and finally reptiles with a single species only identified to group level. No acanthocephalans have been reported in amphibians to date. During the current study 110 frog specimens belonging to 19 species were examined but none harboured any acanthocephalans. However, this forms a small part of the entire amphibian fauna of the country which includes 128 described frog species (Frost 2018).

Only a small fraction of the vertebrate fauna of South Africa has been surveyed for acanthocephalans and we expect that in future additional acanthocephalan species will be discovered and described. For example it is estimated that many of South Africa’s marine fish parasites have yet to be discovered (Smit and Hadfield 2015). South Africa has an extremely rich biodiversity (Huntley et al. 2005), with nearly 8% of the world’s known species of birds, 6% of mammal species and 5% of reptile species (Driver et al. 2012). Therefore we might expect a more diverse acanthocephalan fauna compared to that of Brazil which has 23 genera and 34 species (from only 119 fish species) (Santos et al. 2008) or Iran with 30 described species (Tavakol et al. 2015). The lower species richness reported for South Africa probably reflects sampling effort rather than the true diversity of the acanthocephalan fauna. Until more data are available it will not be possible to determine the true species richness of the South African acanthocephalan assemblage.

## Supplementary Material

XML Treatment for Mediorhynchus africanus

XML Treatment for Mediorhynchus mokgalongi

XML Treatment for Mediorhynchus numidae

XML Treatment for Mediorhynchus taeniatus

XML Treatment for Moniliformis kalahariensis

XML Treatment for Moniliformis moniliformis

XML Treatment for Moniliformis acomysi

XML Treatment for Moniliformis

XML Treatment for Heptamegacanthus niekerki

XML Treatment for Oligacanthorhynchidae

XML Treatment for Acanthogyrus (Acanthosentis) phillipi

XML Treatment for Acanthogyrus

XML Treatment for Neoechinorhynchus (Neoechinorhynchus) dorsovaginatus

XML Treatment for Longicollum chabanaudi

XML Treatment for Longicollum

XML Treatment for Rhadinorhynchus cadenati

XML Treatment for Rhadinorhynchus capensis

XML Treatment for Rhadinorhynchus

XML Treatment for Centrorhynchus sarehae

XML Treatment for Centrorhynchus clitorideus

XML Treatment for Centrorhynchus

XML Treatment for Centrorhynchus

XML Treatment for Plagiorhynchus (Prosthorhynchus) cylindraceus

XML Treatment for Andracantha tunitae

XML Treatment for Arhythmorhynchus turbidus

XML Treatment for Bolbosoma capitatum

XML Treatment for Bolbosoma vasculosum

XML Treatment for Corynosoma australe
